# Increasing access to comprehensive care for Chagas disease: development of a patient-centered model in Colombia

**DOI:** 10.26633/RPSP.2017.153

**Published:** 2017-12-05

**Authors:** Andrea Marchiol, Colin Forsyth, Oscar Bernal, Carlos Valencia Hernández, Zulma Cucunubá, Eduin Pachón Abril, Mauricio Javier Vera Soto, Carolina Batista

**Affiliations:** 1 Drugs for Neglected Diseases initiative Latin America Drugs for Neglected Diseases initiative Latin America Rio de Janeiro Brazil Drugs for Neglected Diseases initiative Latin America, Rio de Janeiro, Brazil.; 2 Red Chagas, Grupo de Parasitología Red Chagas, Grupo de Parasitología Bogotá Colombia Red Chagas, Grupo de Parasitología, Instituto Nacional de Salud, Bogotá, Colombia.; 3 Ministry of Health and Social Protection Republic of Colombia Bogotá Colombia Ministry of Health and Social Protection, Republic of Colombia, Bogotá, Colombia.

**Keywords:** Chagas disease, *Trypanosoma cruzi*, neglected diseases, health systems, quality of health care, Colombia, Enfermedad de Chagas, *Trypanosoma cruzi*, enfermedades desatendidas, sistemas de salud, calidad de la atención de salud, Colombia, Doença de Chagas, *Trypanosoma cruzi*, doenças negligenciadas, sistemas de saúde, qualidade da assistência à saúde, Colômbia

## Abstract

*Worldwide, over 6 million people are infected with* Trypanosoma cruzi, *the pathogen that causes Chagas disease (CD). In the Americas, CD creates the greatest burden in disability-adjusted life years of any parasitic infection. In Colombia, 437 000 people are infected with* T. cruzi, *of whom 131 000 suffer from cardiomyopathy. Colombia’s annual costs for treating patients with advanced CD reach US$ 175 016 000. Although timely etiological treatment can significantly delay or prevent development of cardiomyopathy—and costs just US$ 30 per patient—fewer than 1% of people with CD in Colombia and elsewhere receive it. This represents a missed opportunity for increasing patients’ healthy, productive years of life while significantly reducing the economic burden on the health care system. Key barriers are complexities and delays in the diagnosis and treatment process, lack of awareness of CD among both patients and health care professionals, and administrative barriers at the primary care level*.

*In 2015, stakeholders from government, academia, nongovernmental organizations, and patient associations participated in a seminar in the city of Bogotá on eliminating barriers to diagnosis and treatment for CD. The seminar gave birth to a model of care for increasing patient access, including a patient road map that simplifies diagnostic and treatment processes, shifting them from specialists to primary care facilities. The patient road map was implemented in a pilot project in four endemic communities beginning in 2016, with the goal of testing and refining the model so it can be implemented nationally. This article describes key components in the development of a new, recently implemented model of care for CD in Colombia*.

## RESUMEN

En todo el mundo, hay más de 6 millones de personas infectadas por el *Trypanosoma cruzi*, el agente patógeno causante de la enfermedad de Chagas. En la Región de las Américas, esta es la infección parasitaria que tiene la mayor carga en cuanto a años de vida ajustados en función de la discapacidad. En Colombia, 437 000 personas están infectadas por el *T. cruzi*; de ese total, 131 000 sufren de miocardiopatía. En ese país, el costo anual de tratar a los pacientes que tienen la enfermedad de Chagas se ubica en US$ 175 016 000. A pesar de que un tratamiento etiológico oportuno puede retrasar o prevenir significativamente la aparición de una miocardiopatía —a un costo de apenas US$ 30 por paciente— menos de 1% de las personas con enfermedad de Chagas en Colombia y otros países lo reciben. Esto implica que se pierde la oportunidad de incrementar el número de años de vida saludables y productivos de los pacientes y, al mismo tiempo, reducir significativamente la carga económica que soporta el sistema de atención de salud. Los obstáculos clave son la complejidad y las demoras en los procesos de diagnóstico y tratamiento, la falta de conocimiento sobre la enfermedad de Chagas por parte de los pacientes y de los profesionales de la salud, y las barreras administrativas que existen a nivel de la atención primaria.

En el 2015, representantes del gobierno, la comunidad académica, organizaciones no gubernamentales y asociaciones de pacientes participaron en un seminario en Bogotá sobre la eliminación de las barreras al diagnóstico y el tratamiento de la enfermedad de Chagas. En este seminario se elaboró un modelo de atención para aumentar el acceso de los pacientes, incluida una hoja de ruta centrada en el paciente que simplifica los procesos de diagnóstico y tratamiento al trasladarlos de los especialistas a los establecimientos de atención primaria. La hoja de ruta centrada en el paciente se aplicó a principios del 2016 como parte de un proyecto piloto que se puso en marcha en cuatro comunidades endémicas con el objetivo de poner a prueba y perfeccionar el modelo para luego poder aplicarlo en todo el país. En este artículo se describen los componentes clave que se usaron para crear un modelo de atención de la enfermedad de Chagas puesto en marcha recientemente en Colombia.

## RESUMO

*Em* todo o mundo, cerca de 6 milhões de pessoas são infectadas pelo *Trypanosoma cruzi*, o patógeno causador da doença de Chagas. Nas Américas, esta infecção parasitária é responsável pela maior carga de anos de vida perdidos ajustados por incapacidade. Na Colômbia, estima-se que 437 mil pessoas são infectadas pelo *T. cruzi*, das quais 131 mil têm miocardiopatia. O custo anual para tratar os pacientes com doença de Chagas em estágio avançado chega a US$ 175.016.000. Embora o tratamento oportuno mirando o agente etiológico possa postergar significativamente, ou prevenir, a ocorrência de miocardiopatia, ao custo de apenas US$ 30 por paciente, menos de 1% dos portadores da doença de Chagas é tratado na Colômbia e em outros lugares. Representa uma perda de oportunidade de prolongar os anos de vida saudável e produtiva dos pacientes e de reduzir consideravelmente o ônus econômico ao sistema de saúde. Os principais entraves são a complexidade e a demora do processo de diagnóstico e tratamento, a falta de conhecimento sobre a doença por parte dos pacientes e dos profissionais da saúde e os obstáculos administrativos ao nível da atenção primária. ***Em*** 2015, interessados diretos de setores do governo, comunidade acadêmica, organizações não governamentais e associações de pacientes participaram de um seminário realizado na cidade de Bogotá em que discutiram como eliminar as barreiras ao diagnóstico e ao tratamento da doença de Chagas. Deste seminário nasceu um modelo para aumentar o acesso dos pacientes à atenção à saúde, com a preparação de um guia simplificado para o diagnóstico e o tratamento e a transição do atendimento dos serviços especializados aos serviços de atenção primária. Como parte de um projeto-piloto, a partir de 2016, o guia do paciente foi implantado em quatro comunidades endêmicas com o propósito de testar e aprimorar o modelo para que possa ser implantado em todo o país. O presente artigo descreve os principais componentes deste modelo de atenção para a doença de Chagas recém-implantado na Colômbia.

Worldwide, over 6 million people are infected with *Trypanosoma cruzi*, the protozoan that causes Chagas disease (CD),yet fewer than 1% of them receive treatment. In Colombia, CD poses a major public health challenge. An estimated 437 000 Colombians are infected with *T. cruzi*, of whom 131 000 suffer from cardiomyopathy due to the advancement of the disease (1). CD also creates a heavy economic burden; the estimated annual cost of treating a patient in the chronic stage of the disease was US$ 1 028 in 2004 (equivalent to US$ 1 336 in 2017) (2). This entails a total annual cost of US$ 175 016 000 (2017 dollars) for Colombia’s health system.

CD is most often transmitted by insects of the triatomine subfamily, also known as kissing bugs or, in Colombia, *pitos*. However, CD can also spread via blood transfusion, organ transplant, or congenital transmission. Additionally, oral transmission through food contaminated by triatomine feces or contact with infected reservoir species is an emerging public health issue in Colombia and other Latin American countries (3–6).

CD consists of an acute stage followed by a lengthy asymptomatic period. Years after the acute phase, 30%-40% of those infected develop severe complications, usually cardiomyopathy (7). Antitrypanosomal treatment with benznidazole or nifurtimox can halt or delay the onset of cardiomyopathy due to CD (8, 9), prevent future congenital transmission (10-12), and achieve sustained parasite clearance (13). However, this treatment, at least when administered according to current guidelines, has not proven effective in patients who have already developed CD cardiomyopathy (14), making it essential to treat patients while they are still in the asymptomatic phase of the disease. The cost of providing screening, diagnosis, and antitrypanosomal treatment is as low as US$ 30 per patient, according to Médecins sans Frontières/Doctors without Borders Bolivia. That represents a substantial savings compared to the annual cost of treating a CD patient with cardiomyopathy.

Nonetheless, most people with CD are undiagnosed and unaware of their infection, and even patients who are diagnosed often encounter significant difficulties in securing treatment. Since the development of Colombia’s National Chagas Control Program in 2008, only 1.2% of the at-risk population has been screened (primarily through blood banks, some community screening efforts, and patient request), and fewer than 0.4% of expected cases have received etiological treatment (15). This article describes the development of a pilot project in Colombia designed to increase patient access to diagnosis and treatment of CD. This model is currently being implemented in four municipalities where CD is endemic (Supplemental Figure).

## ANTECEDENTS TO THE DEVELOPMENT OF COLOMBIA’S MODEL OF CARE FOR CHAGAS DISEASE

Colombia has taken major strides to ensure health care access for all citizens, including marginalized groups who disproportionately suffer from neglected tropical diseases. Health expenditures increased from 5.4% to 6.5% of gross domestic product between 2004 and 2011, and, by 2012, over 90% of the country’s population had insurance coverage (16, 17). Passed in early 2015, Law 1751 presents health as a fundamental right of Colombian citizens and aims to address social inequalities impacting health care. In 2013, Colombia became the first country in the world to receive verification from the World Health Organization of the elimination of onchocerciasis (river blindness). This provided an impetus for efforts to eliminate other neglected tropical diseases in Colombia, including CD. Colombia’s Strategy for the Integrated Management of Health Promotion, Prevention, and Control of Vector-Transmitted Diseases (Estrategia de Gestión Integrada para la Promoción, Prevención y Control de las Enfermedades Transmitidas por Vectores) for 2012-2021 seeks to reduce by 40% the “social and economic burden due to high morbidity, mortality, disability, and complications caused by vector-borne diseases”(18). Colombia’s current 10-Year Public Health Plan (Plan Decenal de Salud Pública) calls for certification of elimination of CD vector transmission in 40% of endemic municipalities (with the remaining 60% in process of certification) by 2021, with a concomitant 30% reduction in mortality from acute CD (19). In 2014, interruption of transmission was certified in 10 municipalities in endemic areas (20).

Despite Colombia’s progress in eliminating transmission of *T. cruzi*, substantial gaps remain in providing diagnosis and care for people living with the infection. In 2014, Colombia’s clinical guidelines for CD, originally formulated in 2010, were revised to be more relevant for primary care settings. Colombia’s Red Chagas (Chagas Network), a collection of actors from government, nongovernmental organizations (NGOs), academia, and patient organizations, focused on generating knowledge and developing strategies to increase access to treatment for CD (unfortunately, the Red Chagas has currently suspended its operations due to lack of financial support).

In 2015, the Ministry of Health and Social Protection (Ministerio de Salud y Protección Social, MSPS), the National Health Institute (Instituto Nacional de Salud, INS), and the Chagas Network, in collaboration with the Drugs for Neglected Diseases *initiative* (DND*i*), organized a seminar titled “Towards the Elimination of Barriers to Access to the Diagnosis and Treatment of Chagas Disease” [Hacia la Eliminación de las Barreras en el Acceso al Diagnóstico y Tratamiento de la Enfermedad de Chagas]. The meeting took place in Bogotá and brought together experts, local public health officials, and patient groups. They proposed creating a patient-centered road map to eliminate barriers and facilitate access to treatment and diagnosis of CD. Specific recommendations from the seminar included: update and official publication of the 2014 CD treatment guidelines; development of an integrated model of care for CD; training/capacity-building of health care personnel to provide CD diagnosis and treatment at the primary care level; expansion of CD health education in atrisk communities; and creation of a national entity for managing critical CD supplies and medications. Initially, the process would be tested through a pilot program, then perfected and officially implemented nationwide.

## BARRIERS TO ACCESS FOR CHAGAS DISEASE TREATMENT IN COLOMBIA

CD is highly correlated with poverty and disproportionately affects the poor in Latin America, especially in rural areas (21). Colombia’s prolonged armed conflict has had a profound political, economic, and social impact, which, in turn, has affected the epidemiology and control of CD. In recent decades, the armed conflict, in combination with economic hardship, forced much of the rural population to seek refuge in major cities. Thus in Colombia, as elsewhere in Latin America, CD has increasingly become an urban phenomenon. Moreover, vector control and medical care in some areas was impeded by the conflict, while military personnel and others serving in the zones of conflict were exposed to the vector. Finally, the palm oil boom and the strong presence of hydrocarbon drilling in areas affected by the conflict have created profound ecological changes, which could favor the spread or reemergence of the vector (22, 23).

The 2015 Bogotá seminar provided a strategic entry point for developing a new collaborative model to increase access to health care for CD. Seminar participants identified various barriers to diagnosis and treatment of CD in Colombia (Table 1). The principal impediments are: 1) delays and loss to follow-up during the diagnostic process; 2) absence of diagnosis and treatment at the primary care level; 3) lack of expertise and training in CD among health care personnel; 4) bureaucratic delays involved in diagnostic confirmation and authorization of treatment; and 5) stipulation in treatment guidelines that only specialists provide CD treatment, which effectively excludes primary care physicians who are more accessible to patients. Proactive screening of CD only occurs sporadically, and there are shortages of diagnostic equipment and supplies in primary health care centers. A confirmatory diagnosis, required in order to initiate treatment, often involves sending samples to distant central reference laboratories, leading to delays of several months.

The medications for treating CD are not registered in Colombia and cannot be purchased directly from the manufacturer. This situation creates substantial barriers in the distribution process of CD medications. Benznidazole and nifurtimox reach Colombia through the Pan American Health Organization (PAHO), which maintains a supply of essential medications. Nifurtimox is provided to the World Health Organization (WHO)— which distributes the medication to PAHO—through an agreement with Bayer, which donates a million tablets annually. Although Colombia generally acquires sufficient stock through this arrangement, there have been disruptions in the supply of benznidazole, and only one manufacturer (ELEA, in Argentina) maintains regular production (24). Moreover, once the drugs are distributed from PAHO to the central Government, bureaucratic processes involved in the distribution of the drugs from the national to departmental and municipal levels create significant delays. Consequently, it may take several months for medications from the central level of the MSPS to reach departmental authorities and eventually primary health centers, at which point the medications may have already expired (15). Therefore, benznidazole is often unavailable in endemic areas, so treatment is more commonly provided with nifurtimox, which produces more frequent side effects (25).

Colombia’s Comprehensive Health Care Model (Modelo Integrado de Atención en Salud) consists of primary care and complementary providers. Primary care facilities are located throughout the country and provide basic care, whereas more specialized or complex services are offered through complementary centers. Although primary care facilities are more accessible to patients, treatment for CD is considered a specialized consultation, which is available mainly at complementary facilities. This, plus the multiple patient visits required to diagnose and treat CD, drives up costs. Meanwhile, providers must fulfill extensive bureaucratic requirements. Many physicians and health care personnel are unaware of current guidelines for diagnosing and treating CD or do not believe treatment is necessary. Many patients are unfamiliar with Chagas disease, which is not often discussed in the media or health education initiatives. There is a need for a process for counseling patients on treatment options, risks, and benefits, and for guidelines to monitor side effects from the medication, which often cause patients to drop out of treatment.

Insurance coverage also impacts access to care for CD. Colombia has two insurance regimes: subsidized and contributive. Contributive plans are funded through worker/employer contributions, whereas subsidized plans are fully or partly paid by the Government. The subsidized regime covers those who are low income, self-employed, unemployed, or employed in the informal sector. While only 30.2% of people with CD are in the contributive regime, more than twice as many (63.4%) have subsidized coverage (26). Nonetheless, there have been fewer than expected claims from the subsidized regime for treatment of CD, which may reflect barriers encountered by patients covered by the subsidized plan in obtaining diagnostic testing (15). Thus far, most CD treatment has occurred within the contributive regime. However, even contributive plans often have differing guidelines for CD and do not always cover diagnostic confirmation.

## DEVELOPMENT OF A PILOT PROJECT FOR THE COLOMBIAN MODEL OF CARE FOR CHAGAS DISEASE

**FIGURE 1.**
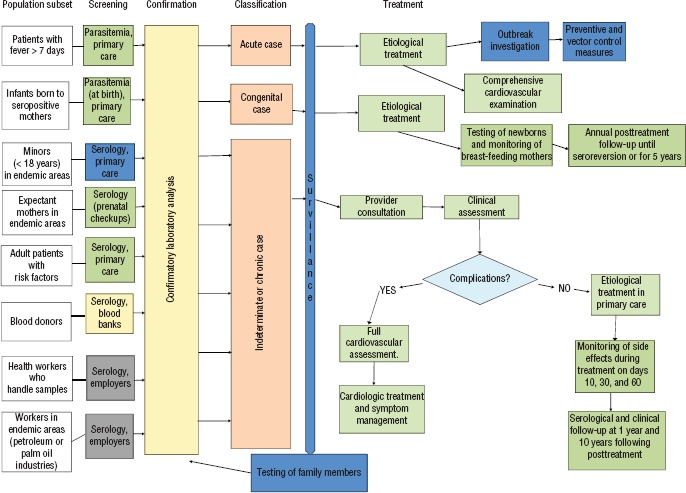
Colombia’s patient road map for Chagas disease, with actions in the public health domain shown in blue

***Source:*** Prepared by the authors from data collected for the study.

Several legislative developments prefaced the creation of a patient-centered road map for CD. Article 65 of Colombia’s Plan Nacional de Desarrollo (National Development Plan) for 2014-2018 calls for creation of comprehensive “patient road maps.” Similarly, Law 1753 prioritizes comprehensive care, Resolution 429 mandates development of patient-centered road maps to guide health care, and Resolution 3202 stipulates creation of a patient-centered road map for CD. The MSPS’s road map for CD has the goal of dramatically improving access to health services, thus increasing levels of treatment and diagnosis in 106 municipalities in endemic areas (out of the country’s 1 122 municipalities). The CD patient road map outlines steps for diagnosing patients, implementing etiological treatment, providing patient education and consultation, managing side effects from the medication, and, if necessary, managing chronic complications, with the bulk of these activities occurring in primary care (Figure 1). The patient road map simplifies the diagnosis and treatment process. For example, for confirmatory diagnosis, the immunofluorescence assay, which requires specialized equipment available only in large laboratories, will be replaced with a less expensive, commercially available enzyme-linked immunosorbent assay (ELISA), which can be more easily processed in or near primary care facilities. This change removes an important barrier to diagnosis and promises to reduce substantially the delay between initial screening results and confirmation, so that treatment can be initiated more rapidly.

**TABLE 1.** Barriers to treatment of Chagas disease (CD) in Colombia, 2015

Diagnosis	Medication	Treatment	Systemic
Long delays for diagnostic confirmation Lack of protocols Lack of risk-based screening tool Lack of supplies and equipment Not available in primary care Delays in sending samples to regional or national laboratories Low public awareness	Delays in importation Delays in distribution from national to regional level Delays in distribution from regional to municipal level Lack of accurate estimates	Distance between rural patients and health centers Numerous patient visits required Low awareness of CD among physicians and patients Bureaucratic delays in authorizing treatment Side effects from medication	Lack of goals and measurement of treatment coverage Clinical guidelines not widely distributed Varying guidelines for CD among insurers Low awareness among insurers of administrative guidelines for managing the medication

***Source:*** Seminar, “Hacia la eliminación de las barreras en el acceso al diagnóstico y tratamiento de enfermedad de Chagas en Colombia,” Bogotá, 22-23 April 2015.

Colombia’s model of care for CD favors decentralization and specifies roles and activities for different levels and actors in the health care system. The MSPS, in addition to providing overall leadership and coordination at the national level, is entrusted with importing benznidazole and other critical medications. The MSPS provides technical assistance to departmental and municipal entities and monitors key indicators. Departmental health programs coordinate actors on a departmental level, ensure medication is efficiently distributed to municipal health centers, assure quality, and monitor results. Both departmental and municipal health entities conduct screening of expectant mothers and minors.

During the model’s development, several potential threats to successful implementation were identified. These included inadequate training of health care personnel, disruptions in the supply chain of benznidazole and nifurtimox, shortages of supplies and personnel, changes in local leadership following elections, and resistance to new processes and guidelines. A key challenge for the MSPS is ensuring proper definition of roles and responsibilities and maintaining clear channels of communication with other branches of government, departmental governments, and NGOs involved in the project.

In order to address these potential threats, test effectiveness in different contexts, anticipate issues, and, if necessary, refine and adjust the model, the MSPS decided to first implement the model of care for CD in a pilot project in select municipalities. This allows for any necessary adjustments so that the model can be smoothly implemented on a national scale. With support from DND*i*, from June to July 2015, several municipalities that manifested their interest were evaluated for inclusion in the pilot project. Public health officials, local authorities, and health care personnel were interviewed in various municipalities in the departments of Arauca, Boyacá, Casanare, and Santander. Criteria for selection included location, availability of material resources and personnel, and political commitment toward providing treatment for CD.

Four municipalities were selected: Soatá, Mogotes, Támara, and Tame. All but Mogotes were certified by PAHO as free of domiciliary vector transmission by *Rhodnius prolixus* in 2014; Mogotes was certified in May 2017 (27). Soatá has a hospital capable of providing specialized care, whereas Támara, Tame, and Mogotes are smaller communities with primary health centers providing basic services, and large proportions of patients with *T. cruzi* infection.

Prior to implementation of the CD patient road map, each of the four municipalities confronted challenges in diagnosing and treating CD (Table 2) (28, 29). Only Soatá could process laboratory samples locally; samples collected in Mogotes were sent to a referral hospital in San Gil, while those from Támara and Tame were sent to their respective departmental capitals or even Bogotá, and processed in various laboratories, depending on patients’ insurance. Delays occurred in soliciting medication from departmental governments, and local stocks were not available in Soatá. Medication in Támara and Tame was only available through the Department of Vector Control, whose staff were trained for entomological rather than clinical activities.

Most patients in these sites lived in rural areas and bore substantial costs both in transportation and missed work in order to go to appointments. To complete the treatment process, patients had to make up to 22 visits to health centers, involving 8 to 11 physician consultations. Health care personnel came primarily from the Mandatory Social Service program, whereby recent graduates of medical or nursing schools must fulfill a year of service in a rural or underserved community. This resulted in frequent turnover, with incoming staff often being unfamiliar with how to provide treatment for CD. The pilot project seeks to address this issue by providing regular training in CD treatment for health care personnel in the four municipalities.

### Goals of the pilot project

The main objective of the pilot project is to validate the model of care for CD developed by the MSPS. The specific project goals are to:

- increase access to diagnosis, treatment, and monitoring for patients with CD
- increase the availability of facilities with technical capacity for treating CD, and ensure adequate training of personnel involved in treating CD
- improve the quality of diagnosis and care for patients with CD
- implement multilevel collaboration in the public health sector to eliminate barriers involved in diagnosing and treating CD

On a more tactical level, the pilot project aims to develop Soatá as a referral site for smaller communities in northern Boyacá department, whereas Tame will fulfill the same role in Arauca. On the other hand, Támara and Mogotes will serve as models for providing treatment for CD at the primary care level. The project aims to eliminate delays by providing diagnostic confirmation within the pilot communities. By simplifying the treatment process and incorporating it into regular primary care, the number of physician consultations required to initiate etiological treatment will decrease from the current level (11) to 4 or fewer. Patients’ initiation and completion of treatment will be systematically tracked. Health care personnel within the pilot region will receive regular biannual training on managing CD, and the MSPS will ensure that a regular supply of benznidazole is available at health centers in the pilot communities.

### Implementation of the pilot project

The comprehensive patient road map for CD was officially approved in July 2016. The pilot project is implementing the road map in one participating municipality at a time, following careful training of health care personnel. Medical personnel from Casanare and Arauca departments completed a CD treatment workshop provided by the Chagas Platform in Bolivia in September 2016, and personnel from Santander and Boyacá departments underwent the same training in May 2017. (The Chagas Platform is an international network of experts and clinicians dedicated to improving medical care for the disease.) Subsequently, additional on-site instruction will taken place. The comprehensive patient road map was implemented in Támara in March 2017, and in Soatá and Mogotes in July 2017. Implementation in Tame is planned for February 2018.

**TABLE 2.** Key health indicators for the four pilot municipalities for the Colombian model of care for Chagas disease

Indicator	Municipality			
	Mogotes	Soatá	Támara	Tame
Population	10 880	7 255	11 881	52 768
Department	Santander	Boyacá	Casanare	Arauca
Departmental ***T. cruzi*** prevalence (%)	6.3	3.7	10.0	21.1
Health center	Primary care (basic consultations)	Complementary (specialist consultations)	Primary care (basic consultations)	Complementary (specialist consultations)
Referral hospital	San Gil	NA^a^	Departmental capital (Yopal)	Departmental capital (Arauca)
ELISA processed locally^b^	No	Yes	No	No
Average delays for diagnosis	4 months	6-8 months	2 months	3 months
Benznidazole stock locally available	Yes	No	Through Department of Vector Control	Through Department of Vector Control
Physicians per 10 000 inhabitants	2	20.6	2.8	0.8
Ratio of publicly subsidized/privately insured patients	5.6:1	1.6:1	3:1	7:1

***Source:*** Prepared by the authors from data collected for the study, including with population according to (28) and departmental T. cruzi prevalence according to (29).

a NA = not applicable (Soatá has a referral hospital within its municipal territory).

b ELISA = enzyme-linked immunosorbent assay.

Health care personnel in the four communities will collect data on the number of patients screened, diagnosed, and treated. Table 3 describes specific measures for evaluation of the project, which will take place in late 2018. Colombia will report on indicators related to comprehensive care of infected individuals at meetings of the Initiative of Andean Countries, PAHO’s subregional collaboration to combat CD in Colombia, Ecuador, Peru, and Venezuela.

## PUBLIC HEALTH IMPLICATIONS

**FIGURE 2.**
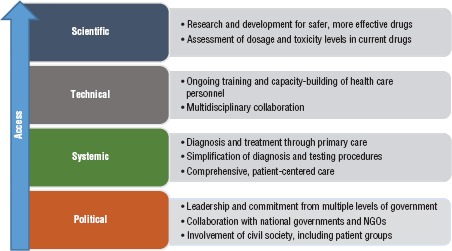
Core ingredients for increasing treatment access for patients with Chagas disease

***Source:*** Prepared by the authors from data collected for the study.

To increase access to health care for people with CD, it is necessary to transform the current paradigm, under which fewer than 1% of people with the infection receive treatment. Many of the challenges involved in treating CD in Colombia are shared by other endemic and even nonendemic countries (30-35). Although local contexts differ, several core components of the Colombian pilot project could provide a framework for increasing access to CD health care in diverse countries (Figure 2). This model emphasizes the role of government and civil society, the elimination of barriers within the health system, maximization of human and technical resources, and the involvement of the scientific community.

The model is predicated on the resolve of government to eliminate diseases of poverty and neglect while expanding access to health care for all segments of society. Policies and legislation lay a crucial groundwork by addressing socioeconomic inequalities and barriers preventing underserved patients from receiving health care. Specifically, the sustainability of the patient road map for CD is supported by Resolution 3202 (2016) from the Ministry of Health and Social Protection, as well as the broader policy of Comprehensive Health Care, by which the national Government established health care as a fundamental human right supported by the State (Resolution 429, 2016). On the local level, the voluntary participation of the municipalities further reinforces the sustainability of the project, and is an indispensable ingredient for success. As in the Colombian example, multiple stakeholders should be involved both in the identification of barriers and in the development of a model of care for CD. This includes multiple levels of government, public health officials, community leaders, health care personnel, patient groups, and domestic and external organizations with expertise, such as the Chagas Network and DND*i*.

On a systemic level, Colombia developed a specific patient road map for CD that addresses the clinical complexities as well as the socioeconomic dimensions of the disease. From a clinical standpoint, the model emphasizes ongoing training of health care personnel in endemic settings, given that these persons often rotate frequently. To address socioeconomic disparities affecting treatment access, the model seeks to decentralize care, moving it from specialists in referral centers to the primary care level in endemic communities.

Finally, the CD patient road map has eliminated many complexities involved in diagnosing and treating CD, reducing the previously high number of patient visits and saving costs for the health care system and patients. However, opportunities remain for further simplifying diagnosis and treatment of CD. Testing with rapid assays would facilitate diagnosis within primary care, and delegation of more of the patient monitoring responsibilities from physicians to nurses would enhance workflow.

**TABLE 3.** Diagnostic and treatment objectives of the pilot project for a Colombian model of care for Chagas disease (CD)

Objective	Measurable outcomes (by Year 3)
1) Increase access to diagnosis,treatment, and monitoring for patients with CD	> 20% of at-risk population screened > 80% of women screened in prenatal care 100% of newborns screened 100% of positive initial diagnoses confirmed following the new diagnostic algorithm > 50% of confirmed cases treated > 50% of treated patients receive annual follow-up
2) Increase the availability of facilities capable of treating CD, and ensure adequate training of personnel involved in treating CD	100% of health centers in pilot area equipped to diagnose and treat CD 100% of health personnel in pilot area trained to manage CD 100% of health centers will have a sustained supply of benznidazole 100% of health centers will have the necessary testing supplies one diagnostic center per municipal network two trainings conducted annually in each municipality defined process in place for patient referrals
3) Improve the quality of diagnosis and care for patients with CD	one trained health care professional in each network, both for treatment and diagnosis 100% of technical requirements for laboratory diagnosis met every diagnosed patient receives a consultation

***Source:*** Prepared by the authors from data collected for the study.

In the Colombian experience, implementation of the model in a pilot project in select municipalities has been an efficient way to test effectiveness and identify potential logistical issues prior to large-scale implementation. The Colombian example provides hope that, with the political commitment of national governments and the involvement of multiple stakeholders, treatment access for CD can be dramatically expanded.

## Acknowledgments.

We are grateful to Colombia’s Ministry of Health and Social Protection, National Health Institute, and the National Chagas Program,as well as the communities participating in the pilot project, for invaluable support in the development of this article and the pilot project it describes. We also wish to thank Jorge Martín and Rafael Herazo from DND*i*, and Carolina Flórez Sánchez and Andrés Caicedo from the National Institute of Health, Colombia, who offered insights and support to strengthen the article. Thanks to Juan Carlos Sequeira for helping with translation of the abstract. Many thanks as well to the anonymous reviewers who provided very helpful feedback and suggestions. Finally, we offer our deepest thanks to the people of Colombia, including those with Chagas disease and those fighting to end the disease’s neglect.

## Funding.

DND*i* is grateful to its donors, public and private, who have provided funding to DND*i* since its inception in 2003. A full list of DND*i*’s donors can be found at http://www.dndi.org/donors/donors/

## Disclaimer.

Authors hold sole responsibility for the views expressed in the manuscript, which may not necessarily reflect the opinion or policy of the *RPSP/PAJPH* and/or PAHO.
